# Keratin 17 identifies the most lethal molecular subtype of pancreatic cancer

**DOI:** 10.1038/s41598-019-47519-4

**Published:** 2019-08-02

**Authors:** Lucia Roa-Peña, Cindy V. Leiton, Sruthi Babu, Chun-Hao Pan, Elizabeth A. Vanner, Ali Akalin, Jela Bandovic, Richard A. Moffitt, Kenneth R. Shroyer, Luisa F. Escobar-Hoyos

**Affiliations:** 1 Department of Pathology, Renaissance School of Medicine, Stony Brook University, Stony Brook, NY 11794 USA; 2 Department of Pathology, School of Medicine, Universidad Nacional de Colombia, Bogotá, Colombia; 3 Department of Family, Population & Preventive Medicine, Renaissance School of Medicine, Stony Brook University, Stony Brook, NY 11794 USA; 4 Molecular and Cellular Biology Graduate Program, Stony Brook University, Stony Brook, NY 11794 USA; 5 Department of Biomedical Informatics, Renaissance School of Medicine, Stony Brook University, Stony Brook, NY 11794 USA; 6 Bascom Palmer Eye Institute, Miller School of Medicine, University of Miami, Miami, FL 33136 USA; 7 Department of Pathology, University of Massachusetts Memorial Medical Center, Worcester, Massachusetts 01655 USA; 8 David M. Rubenstein Center for Pancreatic Cancer Research, Memorial Sloan Kettering Cancer Center, New York, NY 10065 USA; 9 Genetic Toxicology and Cytogenetics Research Group, Department of Biology, School of Natural Sciences and Education, Universidad del Cauca, Popayán, Colombia

**Keywords:** Pancreatic cancer, Prognostic markers

## Abstract

Although the overall five-year survival of patients with pancreatic ductal adenocarcinoma (PDAC) is dismal, there are survival differences between cases with clinically and pathologically indistinguishable characteristics, suggesting that there are uncharacterized properties that drive tumor progression. Recent mRNA sequencing studies reported gene-expression signatures that define PDAC molecular subtypes that correlate with differences in survival. We previously identified Keratin 17 (K17) as a negative prognostic biomarker in other cancer types. Here, we set out to determine if K17 is as accurate as molecular subtyping of PDAC to identify patients with the shortest survival. K17 mRNA was analyzed in two independent PDAC cohorts for discovery (n = 124) and validation (n = 145). Immunohistochemical localization and scoring of K17 immunohistochemistry (IHC) was performed in a third independent cohort (n = 74). Kaplan-Meier and Cox proportional-hazard regression models were analyzed to determine cancer specific survival differences in low vs. high mRNA K17 expressing cases. We established that K17 expression in PDACs defines the most aggressive form of the disease. By using Cox proportional hazard ratio, we found that increased expression of K17 at the IHC level is also associated with decreased survival of PDAC patients. Additionally, within PDACs of advanced stage and negative surgical margins, K17 at both mRNA and IHC level is sufficient to identify the subgroup with the shortest survival. These results identify K17 as a novel negative prognostic biomarker that could inform patient management decisions.

## Introduction

The prognosis of patients with pancreatic ductal adenocarcinoma (PDAC), the most common form of pancreatic cancer, is mainly based on the assessment of the clinical and pathologic presentation at the time of diagnosis^1,2^. While tumor size, lymph node status, metastasis and surgical margins^2^ predict survival, patients with the same clinicopathologic characteristics can have different survival outcomes^3^, suggesting that other undetermined properties of PDAC may drive these differences.

Currently available biomarkers that guide the management of PDAC have limited ability to predict patient clinical course or survival and prior large-scale genomic studies demonstrated that common driver mutations or other mutations lack prognostic value^4–7^. CA 19-9 is the only FDA-approved PDAC biomarker used to determine resectability but is not an accurate predictor of outcome^8–11^. Thus, identification of new prognostic biomarkers to stratify patient subgroups with differential survival probabilities at baseline could be important to guide clinical decision making^12^. Furthermore, understanding the underlying molecular heterogeneity of PDAC may one day guide the development of novel therapeutic strategies.

We discovered that keratin 17 (K17) was a biomarker of aggressive cervical cancers through an unbiased proteomic screen^13^. Several additional studies, including ours, have identified K17 as a negative prognostic biomarker not only in cervical squamous cell carcinoma^13^ but also in endocervical adenocarcinoma^14^, epithelial ovarian carcinoma^15^, triple negative breast carcinoma^16^, gastric adenocarcinoma^17^, gallbladder adenocarcinoma^18^ and head and neck squamous cell carcinoma^19^. Recent RNA sequencing (RNA-Seq) studies^20,21^, including ours^22^, reported that mRNA expression defines molecular subtypes of PDAC including the relatively less aggressive “classical” subtype and the more aggressive “basal-like” subtype that is defined based on the expression of a 25-gene expression signature^20,22^. Embedded within this gene-expression signature, we identified the presence of K17 mRNA. Thus, two different and unbiased approaches, biomarker-based discovery by proteomics and molecular subtyping by RNA-Seq, point to the importance of K17 in PDAC.

Here, we show that K17 identifies PDAC patients with the shortest survival, even in those with the same advanced stage and negative margin status. As detected by immunohistochemistry (IHC), K17 offers a rapid and deployable clinical pathological-assessment technique to guide clinical management at the time of diagnosis. This also represents the first step in the development of a CLIA-certified assay that can be tested in clinical trials and implemented in pathology diagnostic laboratories for PDAC subtyping.

## Results

### Keratin 17 mRNA alone is as accurate as molecular subtyping to predict PDAC prognosis

**Figure 1:**
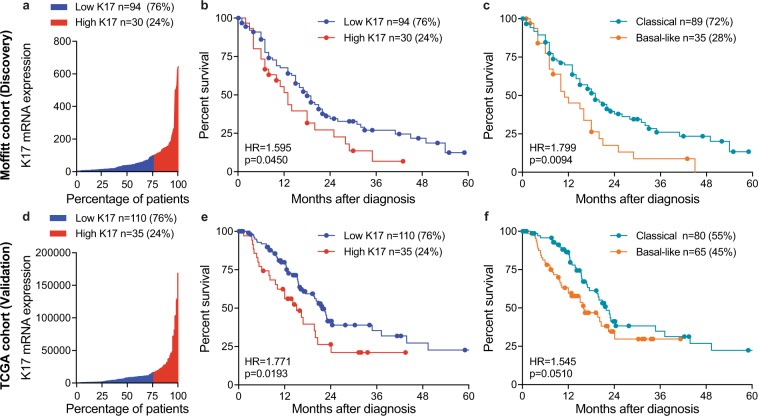
K17 mRNA is as accurate as molecular subtyping to predict prognosis of PDAC. (**a**) Water plot depicts K17 mRNA expression levels in Moffitt *et al*. cohort^22^. 76^th^ percentile defined the cut-off to predict outcome by maximum likelihood fit of a Cox proportional hazard model, 76% of PDAC cases were found to be low K17 (blue) while 24% of cases were classified as high K17 (red). K17 mRNA ranged from 3.559 to 645.377 absolute fluorescence reads. (**b**) Kaplan-Meier curve depicting the overall survival for K17 of resected PDAC primary tumors from Moffitt *et al*. cohort^22^. Cox proportional model was used for analysis. Hazard ratios (HR) and p-values are shown. (**c**) Kaplan-Meier curve of overall survival analysis for mRNA molecular subtypes of resected PDAC primary tumors from Moffitt *et al*. cohort^22^. For analysis, Cox proportional model was used. Hazard ratios (HR) and p-values are shown. (**d**) Water plot depicts K17 mRNA expression levels in The Cancer Genome Atlas (TCGA) cohort^23^. 76th percentile defined the best cut-off to predict outcome by the maximum likelihood fit of a Cox proportional hazard model, 76% of PDAC cases where found to be low-K17 (blue) while 24% of cases were classified as high-K17 (red). K17 mRNA ranged from 75.39 to 170,437.66 RSEM reads. (**e**) Kaplan-Meier curve depicting the overall survival for K17 of resected PDAC primary tumors from TCGA cohort^23^. Cox proportional model was used for analysis. Hazard ratios (HR) and p-values are shown. (**f**) Kaplan-Meier curve of overall survival analysis for mRNA molecular subtypes of resected PDAC primary tumors from TCGA cohort^23^. Cox proportional model was used for analysis. Hazard ratios (HR) and p-values are shown.

Based on our previous findings that K17 is a validated prognostic biomarker in other cancer types^13–19^, we set out to determine its prognostic value in PDAC. Using the Moffitt *et al*.^22^ patient dataset (n = 124) (Table 1), we first analyzed the distribution of K17 mRNA expression across cases. To define a threshold that provided maximal stratification of survival differences based on K17 mRNA, the maximum likelihood fit of a Cox proportional hazard model was used. 76% of the cases were classified as low K17 while 24% of cases were classified as high K17 (Fig. 1a). On average, K17 mRNA expression was 5-times higher in high versus low K17 cases.

**Table 1:** Patient cohort demographics.

	Patient Cohorts		
	mRNA		IHC
	MOFFITT^a^	TCGA^b^	SBU + UMASS^c^
**Months of follow-up, mean ± SD**	13 ± 14	14.1 ± 13.9	17.8 ± 15.2
**Age at diagnosis, mean ± SD**	No information	65.0 ± 11.1	65.5 ± 9.9
**Gender, number (%)**	n = 112	n = 145	n = 74
Female	64 (57%)	66 (46%)	38 (51%)
Male	48 (43%)	79 (54%)	36 (49%)
**Histologic grade (G), number (%)**	No Information	n = 145	n = 74
G1 + G2, Low and Moderate Grade		76 (52%)	42 (57%)
G3, Poor Grade		69 (48%)	32 (43%)
**Surgical Margins (R), number (%)**	n = 104	n = 128	n = 74
R0, Negative margin	66 (63%)	80 (63%)	52 (70%)
R1, Positive margin	38 (37%)	48 (38%)	22 (30%)
**Primary Tumor**^**d**^ **(T), number (%)**	n = 109	n = 144	n = 74
T1 + T2	20 (18%)	21 (15%)	15 (20%)
T3 + T4	89 (82%)	123 (85%)	59 (80%)
**Lymph node status**^d^ **(N), number (%)**	n = 111	n = 144	n = 74
N0, No regional lymph node metastasis	34 (31%)	37 (26%)	28 (38%)
N1, Regional lymph node metastasis	77 (69%)	107 (74%)	46 (62%)
**Cancer Stage**^**d**^**, number (%)**	n = 109	n = 143	n = 74
I-IIA	33 (30%)	34 (24%)	26 (35%)
IIB-IV	76 (70%)	109 (76%)	48 (65%)
**Molecular Subtype, number (%)**	n = 124	n = 145	Not available
Classical	89 (72%)	80 (55%)	
Basal-like	35 (28%)	65 (44%)	
**K17 status, number (%)**	n = 124	n = 145	Not applicable
Low K17	94 (76%)	110 (76%)	
High K17	30 (24%)	35 (24%)	

^a^Moffitt *et al*., Nature Genetics 2015.

^b^TCGA: The Cancer Genome Atlas, Cancer Cell 2017.

^c^SBU: Stony Brook University, UMASS: University of Massachusetts.

^d^Stage classification per AJCC 7^th^ edition.

To determine survival differences between low and high K17 cases, we performed a Kaplan- Meier (K-M) and Cox proportional-hazard regression model of survival and found that patients with high K17 PDACs had a median survival of 13 months, which was shorter than for patients with low K17 PDACs, who had a median survival of 18 months (HR = 1.6, p = 0.0450, CI = 0.935–2.723) (Fig. 1b). To determine the percentage of agreement between the Moffitt molecular subtype and K17 status in the same patient cohort, we performed a chi-square analysis of both K-M curves. The percentage of total agreement between K17 mRNA (Fig. 1b) and molecular subtype (Fig. 1c) K-M curves was 73.4. This showed that out of the 35 cases classified as basal-like subtype, 16 (46%) were also high K17. In addition, out of 89 cases classified as classical subtype, 75 (84%) were also low K17. Thus, the basal-like molecular subtype correlated with high K17 expression in a high proportion of cases. In comparison to the survival probabilities of molecular subtypes reported by Moffitt *et al*.^22^ (HR = 1.8, p = 0.0094, CI = 1.053–3.074) (Fig. 1c), the low versus high K17 classification were statistically similar (Fig. 1b).

To validate the accuracy of K17 mRNA as a negative prognostic biomarker for PDAC in an independent dataset, we employed The Cancer Genome Atlas (TCGA) PDAC database^23^ (n = 145; Table 1). Applying the same maximum likelihood fit of a Cox proportional hazard model threshold identified in the initial mRNA cohort^22^ (Fig. 1d), we found that high K17 mRNA was again a negative prognostic biomarker, with a median survival of 15 months for high K17 and 22 months for low K17 PDACs (HR = 1.8, p = 0.0193, CI = 1.006–3.119) (Fig. 1e). In comparison to basal-like versus classical molecular subtyping (HR = 1.5, p = 0.0510, CI = 0.968–2.467) (Fig. 1f), the negative prognostic accuracy of K17 mRNA status was once again statistically similar. The percentage of agreement by chi-square showed that 26 cases (40%) classified as basal-like subtype were also high K17 and 70 cases (88%) classified as classical subtype were low K17. The percentage of total agreement between K17 mRNA (Fig. 1e) and molecular subtype (Fig. 1f) K-M curves was 66.2. Together, these results indicate that high K17 mRNA status identifies an overlapping subset of patient cases and is as accurate as molecular subtyping in stratifying PDAC patients with the worst outcome in both the TCGA^23^ and the Moffitt *et al*.^22^ cohorts.

### Immunohistochemical expression of K17

**Figure 2:**
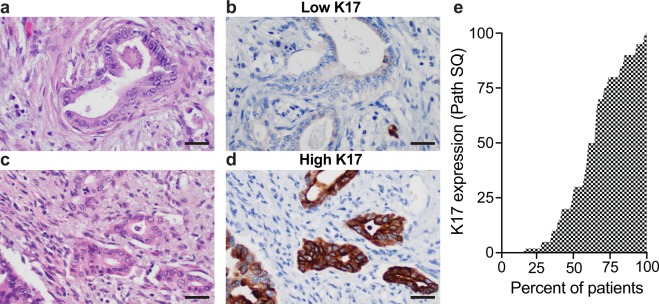
K17 immunohistochemistry in PDAC cases. (**a**–**d**) Representative images from two PDAC cases with similar histologic grade and immunohistochemical stains. Hematoxylin and eosin stained sections (**a**,**c**) and corresponding sections processed for K17 IHC (**b**,**d**). Note similar histologic features of low versus high-K17 expression. Scale bar = 20 μm. (**e**) Water plot depicts K17 IHC score levels in the IHC cohort.

**Figure 3:**
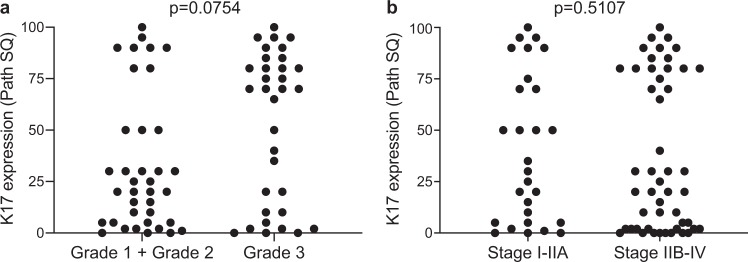
K17 IHC expression is independent of the histologic grade and tumor stage. (**a**) Graph illustrating K17 IHC expression for each case within the same grade category (grade 1 + grade 2 vs grade 3). Path SQ score ranges from 0 to 100% in both categories. P-value was calculated using the Mann Whitney test. (**b**) Graph showing expression of K17 IHC for each case within the same tumor stage category (stage I-IIA vs stage IIB-IV). Path SQ score ranges from 0 to 100% in both categories. P-value was calculated using the Mann Whitney test.

Based on virtual microdissection from bulk-tumor RNA-Seq^22^, we previously predicted that K17 mRNA is derived from tumor cells but not from other benign tumor cellular components. We used IHC to detect K17 at the protein level in 74 formalin-fixed paraffin-embedded (FFPE) PDAC tissue sections, each representing a unique patient (protein cohort; Table 1). K17 protein was identified in the tumor epithelial cells (Fig. 2a–d), in pancreatic intraepithelial neoplasia grade 2 and 3 (PanIN2/3), and was occasionally seen in centroacinar cells but was not detected in PanIN1, benign proliferative ducts, or in other cellular elements, substantially validating our previous *in silico* predictions^22^. To determine the level of expression of K17 by immunohistochemistry in PDAC, we used a semi-quantitative IHC scoring method, the PathSQ score, where the percentage of strong-positively stained tumoral cells on a scale of 0–100% was determined within a single representative histologic section from each case (Fig. 2e)^13^. The range of K17 staining was similar within the same grade or stage of the tumors (Fig. 3a,b). These results show that K17 IHC specifically identifies tumor cells.

### K17 is an independent negative prognostic biomarker at both mRNA and IHC levels and provides additional clinicopathological information

**Figure 4:**
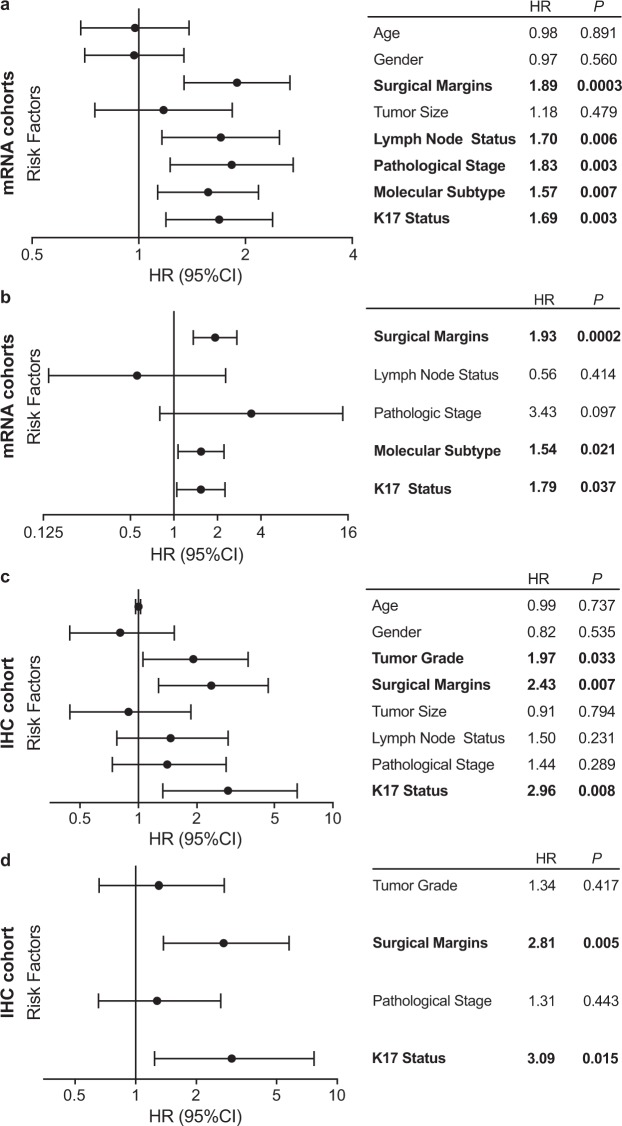
K17 is an independent negative prognostic biomarker, at both the mRNA and IHC (protein) levels. (**a**) Forest plot showing the univariate analysis using Cox proportional hazards regression for K17 mRNA as a binary variable and other PDAC risk factors from combined mRNA cohorts (Moffitt *et al*.^22^ and The Cancer Genome Atlas [TCGA])^23^. Surgical margin status, lymph node status, pathologic stage, molecular subtype and K17 status were all negative prognostic markers with significant p-values. (**b**) Forest plot showing the multivariate analysis from K17 mRNA as a binary variable and other risk factors, from combined mRNA cohorts. Surgical margins, molecular subtype and K17 showed significant p-values. (**c**) Forest plot showing the univariate analysis using Cox proportional hazards regression for K17 as a continuous variable and other PDAC risk factors from the IHC cohort. Tumor grade, surgical margins and K17 showed significant p-values. (**d**) Forest plot showing the multivariate analysis from K17 as a continuous variable and other risk factors, from IHC cohort. K17 and surgical margins show significant p-values.

To evaluate if K17 status is independent of other clinicopathologic features, we performed univariate and multivariate analyses using Cox proportional hazard regression of individual risk factors in the combined mRNA cohort (Fig. 4a,b). K17 mRNA, molecular subtype and surgical margin status were independent negative prognostic biomarkers (Fig. 4b). To determine if K17 IHC for K17 was also prognostic, we used a Cox proportional hazard ratio and found that increased expression of K17 was associated with shorter survival of PDAC patients. (HR = 2.9, p = 0.008, CI = 1.3–6.5/100 units of Path SQ score) (Fig. 4c). Multivariate analysis also showed that K17 and surgical margin status were independent negative prognostic biomarkers (Fig. 4d).

**Figure 5:**
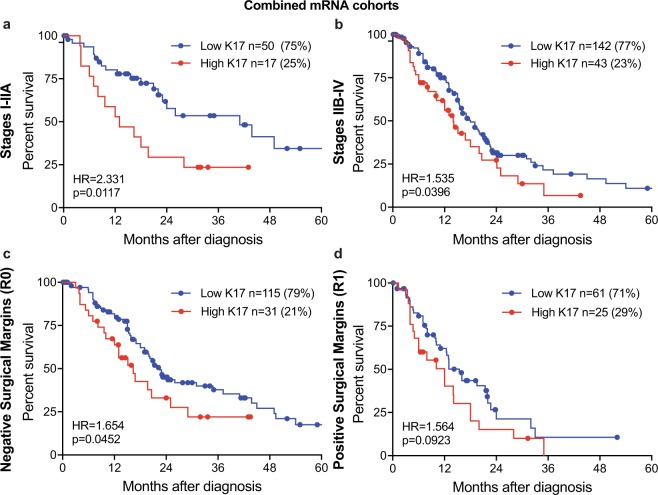
K17 predicts survival based on stage and surgical margins at the mRNA level. (**a–d**) Kaplan–Meier curves depicting the overall survival of the combined mRNA cohorts (Moffitt *et al*.^22^ and The Cancer Genome Atlas [TCGA])^23^ integrating K17 status and tumor stage (**a:** stage I-IIA, **b:** stage IIB-IV) and surgical margins (**c:** negative margins, **d:** positive margins). P-values were calculated using the log-rank test. Hazard ratios (HR) and p-values are shown.

**Figure 6:**
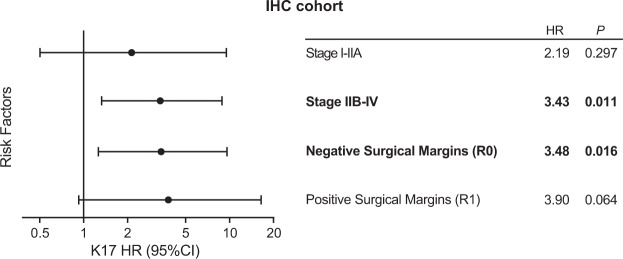
K17 provides additional prognostic value in advance stage and negative margin status groups at the protein level. Forest plot showing interaction of K17 IHC status and tumor stage and surgical margins. Hazard ratios (HR) and p-values are shown. Each HR is computed in subsets of the data.

To determine if K17 provides additive prognostic value in combination with other clinical prognostic factors, we analyzed potential interactions in the mRNA cohorts. K17 mRNA stratified survival differences within patients with localized and advanced disease (Fig. 5a,b) and negative surgical margin status (Fig. 5c) identifying PDACs with the most aggressive clinical course. There was no statistical significance for positive margin status (Fig. 5d). For the IHC cohort, K17 also provided additional information within advanced stage and negative margin group (Fig. 6), as K17 correlated with shorter survival. However, neither K17 mRNA nor IHC data enhanced prognostic value within cases that had positive tumor margins (Figs 5d and 6). Of note, the prognostic significance of K17 was comparable when cases were staged according to 7^th^ and 8^th^ AJCC system.

In summary, K17 is an overall negative independent prognostic biomarker in PDAC and has additional prognostic value within advanced stage PDACs and for patients that had negative surgical margins of resection at both the mRNA and IHC levels.

## Discussion

Here, we demonstrate that K17 is a negative and independent prognostic biomarker at both mRNA, as defined by RNA-Seq, and at the level of protein expression as evaluated by IHC. As a prognostic biomarker, K17 is as accurate as molecular subtyping to predict the patient population with the most aggressive form of the disease.

Currently, there are no methods deployed in clinical practice to predict the differential survival outcome of PDAC patients with indistinguishable clinical and pathological disease characteristics at the time of diagnosis. Through detection and analysis of K17 by IHC in tissues collected at the time of resection, we determined that among patients with the same advanced disease stage, patients with increased expression of K17 have shorter survival. Diagnosis at early stage and negative surgical margin are currently the best clinical factors associated with a higher probability of long-term survival^24^. Keratin 17 independently identifies a subset of patients with worst outcome within advanced stage and negative margin groups. Lower K17 tumor expression may therefore provide a new indicator of the potential PDAC patient long-term survivors. As an independent prognostic biomarker for PDACs, K17 could provide more accurate prediction of prognosis and potentially guide patient management decisions.

We recently identified two PDAC molecular subtypes defined as “classical” and “basal-like” by correlating tumor cell gene expression and patient survival^22^. This classification demonstrates that there are innate biological differences between tumors that can impact treatment decisions and responses. In the survival analysis presented here, molecular subtypes and K17 mRNA status displayed very similar overall survival curves. Although the correlation between molecular subtype and K17 in both cohorts was high, there are clear differences in the separation of the survival curves. There is not a one to one correlation between high-K17 and basal-like subtype and low-K17 and classical subtype, however, we demonstrate that by K17 alone, one of the 25 basal-like subtype genes, we are able to identify patients with the worst prognosis. These findings indicate that the determination of K17 mRNA expression status is as accurate as molecular subtyping as a biomarker of PDACs that have the most aggressive course. Interestingly, there have been reports that other components of the basal-like subtype signature also correlate with poor PDAC prognosis^25–30^. S100A2^27^, AREG^25^, FAM83A^29^, GPR87^28^ and Slc2a1^27^ have all been studied at the IHC level in PDAC tissues and have all been identified as independent prognostic markers, however, the prognostic value of these proteins has not been compared to the molecular subtypes. Further studies will be necessary to understand the role of these proteins in disease biology and response to treatment.

Importantly, recent preliminary results from the COMPASS trial^31^ suggest that the basal-like subtype is less responsive to first-line chemotherapy. Furthermore, GATA6 mRNA identification by *in-situ* hybridization, was identified as a surrogate biomarker of the classical subtype, and prior reports have indicated that patients with GATA6^high^ tumors have better survival than those with GATA6^low^ expression^32^. As expected, GATA6 expression is inversely correlated with that of K17 in both Moffitt *et al*.^22^ dataset and TCGA^23^ database and shows promise as a biomarker of the more favorable form of the disease, predicting survival and response to chemotherapy. The availability of more than one biomarker to subtype patients may be useful in improving prognostic accuracy and identification of the best candidates for novel targeted therapies. Although keratin 81(K81) has also been correlated with shorter survival in two different cohorts (HR = 1.036 and 1.730), K17 status (HR = 2.9) provides a higher HR value. It is also unknown whether K81 is prognostic in advanced stage PDAC or is impacted by surgical margin status^33^. Future work is required to continue understanding the molecular differences between the PDAC subtypes with the aim of improving treatment options.

Previous reports show that K17 mRNA is highly transcribed in PDAC^34,35^ and that at least 30% of cases can be detected by K17 IHC in neoplastic cells^36,37^. One of the most important advantages of IHC is that this method enables interrogation of biomarker expression at the histologic level while RNA-Seq or other methods based on tissue solubilization dilute cancer-specific biomarkers by components derived from other benign cellular elements, including stromal cells, inflammatory cells and benign ductal cells, acinar cells, islet cells, or other benign cellular elements. Thus, IHC enables the determination of K17 status, even in cases that have low tumor cellularity. IHC is also a clinically well established and deployable assay, widely used by pathology laboratories as a routine diagnostic test platform. Here, we demonstrate the prognostic value of K17 IHC test in a small cohort of PDAC patients. Future studies should validate these findings and evaluate the predictive value of K17 IHC testing in retrospective analyses using cohorts of PDAC cases involved in precision medicine testing, potentially including the Pancreatic Cancer Action Network “Know Your Tumor” initiative^38^, among others. Of note, less than 20% of PDAC patients are eligible for surgical resection but in many of those cases, the diagnosis is confirmed only by fine needle aspiration (FNA) biopsy. Thus, further studies are indicated to determine if immunocytochemical scoring of K17 status in FNA specimens could also be used to define PDAC K17 status in surgically unresectable PDACs, as a future prognostic strategy and to help guide decisions regarding selection of options for therapeutic intervention.

In summary, we demonstrate that K17 mRNA is as accurate as molecular subtyping to predict PDAC prognosis. By either RNA-Seq or IHC localization, K17 predicts survival, independent of tumor grade, stage, and margin status. In conclusion, IHC is an optimal platform to clinically deploy K17 as a prognostic biomarker for PDAC.

## Methods

### Patient demographics

Three independent patient cohorts were analyzed for K17 expression, of which two cohorts were analyzed for K17 mRNA sequencing and one for K17 immunohistochemical analysis (Table 1). For our K17 mRNA-1 cohort, we used the same cohort that was reported by Moffitt *et al*.^22^ (n = 124). For the K17 mRNA-2 cohort, we analyzed the TCGA^23^ database patient samples (n = 145). For the K17 IHC analysis, formalin-fixed paraffin-embedded (FFPE) surgical tissue blocks from consecutive PDAC cases from a five-year interval (2008–2012) were selected from the archival collections of the Departments of Pathology at Stony Brook Medicine (n = 56) and the University of Massachusetts (n = 18). The patient selection criteria were diagnosis of primary PDAC and patients over the age of 18 years old at the time of diagnosis. Patients with survival of less than a month after surgery or a diagnosis of cancer metastatic to the pancreas from other anatomic sites were excluded. Survival and adjuvant therapy data was obtained from the respective institution’s registry. All studies were performed in accordance with guidelines and regulations of the Stony Brook Medicine Institutional Review Board (IRB) protocol 94651-36 and University of Massachusetts IRB protocol H00015796. Patient written consent was waived by the IRB of both institutional IRBs because the research was restricted to the analysis of de-identified remnant waste surgical pathology specimens that were provided by each institution.

Patients were stratified based on the American Joint Committee on Cancer (AJCC) 7^th^ edition staging criteria, as recorded in the original surgical pathology diagnostic reports. Tumor size categories were grouped as tumor limited to the pancreas (T1-T2) and tumor that extended beyond the pancreas (T3-T4); lymph node status was recorded as no regional metastases (N0) versus regional metastases (N1); cancer stage was grouped according to tumor limited to the pancreas with no regional or distant lymph node metastases (IA-IIA) versus tumor that extended beyond pancreas and/or was metastatic (IIB-IV). Histological grades were grouped into well and moderately differentiated (G1-G2) versus poorly differentiated (G3). Surgical margins were scored as negative (R0) versus positive (R1) for carcinoma. All analyses were performed in accordance with these criteria.

### Bioinformatic analysis of K17 mRNA expression across databases

We screened the Moffitt *et al*.^22^ and the TCGA^23^ PDAC databases for K17 mRNA expression levels^13^. For the Moffitt *et al*.^22^ dataset, there were 124 cases with survival information. For the TCGA database, 145/150 cases had both RNA Sequencing data (RNASeq V2 RSEM) and survival data. K17 mRNA ranged from 3.559 to 645.377 absolute fluorescence reads in the Moffitt *et al*.^22^ dataset and from 75.39 to 170,437.66 RSEM reads in the TCGA database. Based on the maximum likelihood fit of a Cox proportional hazard model, we selected the 76th percentile of mRNA expression at which to split samples into K17 high or K17 low groups. This threshold was trained on the University of North Carolina^22^ cohort, and subsequently fixed before application to the TCGA^23^ cohort of cases.

### K17 immunohistochemistry

All tumor sections were reviewed from each case to identify the single tissue block for immunohistochemical studies that contained the greatest total surface area of viable carcinoma. Exclusion criteria included cases where the total surface area of viable tumor was <1 cm^2^. An indirect immunoperoxidase method was used to identify the presence of K17 protein, as previously described^13^. Briefly, after incubation at 60 °C, slices were deparaffinized in xylene and rehydrated in alcohols. Antigen retrieval was performed in citrate buffer at 120 °C for 10 minutes in a decloaking chamber. Endogenous peroxidase was blocked by 3% hydrogen peroxide and sections were incubated overnight at 4 °C with: mouse monoclonal-[E3] anti-human K17 antibody (Abcam, Cambridge, MA). After primary antibody, biotinylated horse secondary antibodies (R.T.U. Vectastain ABC kit; Vector Laboratories, Burlingame, CA) were added. Development was done with 3, 3′ diaminobenzidine (DAB) (Dako, Carpinteria, CA) and counter-staining was done with hematoxylin. Negative controls were performed on all runs using an equivalent concentration of a subclass-matched immunoglobulin. K17 staining intensity was classified by one pathologist (K.R.S) based on a subjective assessment of absent (0), light (+1), or strong (+2) staining. The overall proportion of tumor cells with 2+ intensity staining (the PathSQ score) was determined by review of the representative immunostained histologic section from each case, blinded to corresponding clinical data.

### Statistical analyses

The clinicopathologic features were categorized into meaningful groups, hence chi-square test and Fisher’s exact test were used to analyze associations between two categorical variables. The best cutoff point was chosen according to the lowest Akaike’s information criterion from a Cox proportional-hazard regression model to determine low versus high K17 mRNA expression. A data-driven cut off point classified patients into two groups: high K17 (24% of cases) and low K17 (76% of cases). The same cut off point was validated in the TCGA database which showed 24.8% of cases expression high K17 and 75.2% of cases expressing low K17. Overall survival was calculated from the date of diagnosis to the date of death and was estimated using the Kaplan–Meier method, using median or rate at specific time points with 95% confidence interval. Alive patients were censored at the last follow-up; all prognostic analyses were performed as described by Ballman^39^. Univariate analyses compared survival for K17 level (low vs. high), histological grade, tumor size, node status, tumor stage and surgical margins. To examine survival rates while adjusting for potential confounders, multivariate analyses were performed by Cox proportional hazards regression.

For the IHC analysis, K17 Path SQ score was used as a continuous variable in Cox proportional hazard model to determine the survival differences. Similarly, univariate and multivariate analyses compared survival for K17 level of expression, histological grade, tumor size, node status, tumor stage and surgical margins by Cox proportional hazards regression. Statistical significance was set at p-value ≤ 0.05 and analysis was done using SAS 9.4 (SAS Institute, Cary, NC, USA) and Graph pad prism 7 (Graph Pad Software, La Jolla, CA, USA).

### Patent

Shroyer, K, Escobar-Hoyos, L.F. Keratin 17 as a Prognostic and Predictive Marker of Pancreatic Cancer. Stony Brook University Research Foundation. Filed 10/29/15. Pending Provisional Patent Application.
